# Exploring Uncharted Waters: The Role of Artificial Aquatic Ecosystems in Freshwater Science

**DOI:** 10.1002/ece3.73777

**Published:** 2026-06-03

**Authors:** Jackie Webb, Ellen Moon, Julie C. Fahy, Shashini Fernando, Martino E. Malerba, Laura Naslund, Mike Peacock, Naomi S. Wells, Benjamin J. Woolcock, Alexander J. Reisinger

**Affiliations:** ^1^ School of Science, Engineering and Digital Technologies University of Southern Queensland Toowoomba Queensland Australia; ^2^ Centre for Sustainable Agricultural Systems University of Southern Queensland Toowoomba Queensland Australia; ^3^ Centre for Agricultural Engineering University of Southern Queensland Toowoomba Queensland Australia; ^4^ School of Engineering Deakin University Geelong Victoria Australia; ^5^ School of Engineering, Architecture and Landscape of Geneva (HEPIA) HES‐SO—University of Applied Sciences and Arts Western Switzerland Jussy Switzerland; ^6^ Centre for Nature Positive Solutions, Department of Biology, School of Science RMIT University Melbourne Victoria Australia; ^7^ School of Life and Environmental Sciences Deakin University Warrnambool and Burwood Victoria Australia; ^8^ Odum School of Ecology University of Georgia Athens Georgia USA; ^9^ Department of Geography and Planning, School of Environmental Sciences University of Liverpool Liverpool UK; ^10^ Department of Aquatic Sciences and Assessment Swedish University of Agricultural Sciences Uppsala Sweden; ^11^ Department of Soil and Physical Sciences Lincoln University Canterbury New Zealand; ^12^ Department of Soil, Water, and Ecosystem Sciences University of Florida Gainesville Florida USA

**Keywords:** ditches, drainage, farm dams, inland waters, ponds

## Abstract

Artificial aquatic ecosystems (AAEs) are human‐made environments in managed landscapes that encompass a spectrum from straightened streams to newly constructed ponds. Research on AAEs is now expanding into diverse environmental science fields in response to ongoing pressures threatening freshwater ecosystems. Yet AAEs remain sidelined in the traditional study of freshwaters, limiting their recognition in catchment and atmospheric processes. We advocate for increased recognition of AAEs in freshwater science on four key points: (1) their ubiquity across human‐impacted landscapes; (2) their contributions to biogeochemical cycles and ecosystem services; (3) as sources of insights to fundamental questions on pressures facing freshwater ecosystems; and (4) their lack of inclusion in policy. We call for a paradigm shift from viewing AAEs as low in the hierarchy of natural sciences to recognizing them as critical components of the modern hydroscape that offer opportunities for interdisciplinary research and improvement of water resources.

## Graphical abstract

The world's farms and cities house an abundance of human‐made waters, including farm dams, stormwater ponds, constructed wetlands, and ditches: known collectively as “artificial aquatic ecosystems.” In this viewpoint we challenge the entrenched idea that human‐made waters have limited ecological value, discuss the disconnect between the functioning of AAEs and natural aquatic ecosystems, and call attention to how recognizing AAE's in fundamental research will improve freshwater resource management in an increasingly human‐impacted future. To conclude, we suggest a framework that explores solutions to the gaps and opportunities that better integrates AAE's into freshwater science research and policy.

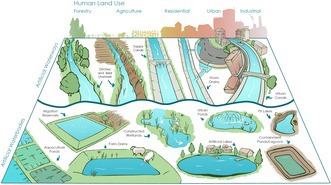

## 1 Introduction

Human activities have intentionally and unintentionally altered the distribution and movement of water across the landscape, fundamentally altering the hydrological cycle (Abbott et al. 2019; Tank et al. 2021). Despite this history of hydrologic alterations, most of our knowledge of the functioning of freshwater ecosystems has developed from ecological theory around natural lakes, streams, rivers, and wetlands (Wetzel 2001; Dodds and Whiles 2010). Agricultural and urban development have altered 40% of the Earth's surface, changing how humans interact with, maintain, and/or exploit water resources (Ellis et al. 2010). The alteration of landscapes for human activities has given rise to artificial aquatic ecosystems (AAEs; Clifford and Heffernan 2018), constructed by humans to provide a range of functions and services.

The ubiquity of AAEs has been recognized across a range of landscapes and regions (Clifford and Heffernan 2018; Koschorreck et al. 2020; Palta et al. 2017; Sinclair et al. 2020). Their proliferation is driven by the myriad demands that society places on water resources. For example, tile drains underlying agricultural fields throughout the midwestern US were installed in the 19th century to drain excess water from the land (Tank et al. 2021). The increasing extent of impervious surface cover in cities (“urban creep”) has also shifted the balance of surface runoff and groundwater recharge, necessitating the management of excessive surface runoff through a variety of means including pipes, ditches, swales, and retention basins. These novel environments represent the modern hydroscape of the Anthropocene. Enhancing our understanding of how and why AAEs function represents an opportunity to further our fundamental understanding of how aquatic ecosystems respond to diverse anthropogenic stressors, while also developing applied solutions to better manage these human‐dominated ecosystems.

There is increasing recognition of the potential for additional ecological services provided by AAEs and other nature‐based solutions (Keeler et al. 2019), but there remains a disconnect between the prevalence of AAEs, our understanding of their functioning, and our ability to manage them for both societal and environmental benefits. Given their central position and abundance within agricultural, forestry, and urban landscapes, AAEs likely play an important yet overlooked role in local, regional, and global biogeochemical cycles, and in addressing societal challenges including pollutant removal and water security (Frost et al. 2015; Peacock et al. 2021a, 2021b).

In this review, we advocate for expanding research on AAEs in freshwater science. First, we explore the concept of artificiality and demonstrate a bias in freshwater research towards natural waters through current trends in the AAE literature. We then highlight the abundance of AAEs, challenge the entrenched idea that human‐made waterbodies have limited value as ecosystems, and assert that recognizing AAEs in policy and fundamental research will improve freshwater resource management in an increasingly human‐impacted future. We examine these points using examples of common AAE types in agricultural and urban landscapes and conclude with directions to help advance these efforts.

## 2 What Makes an Aquatic Ecosystem “Artificial”—and Why Should We Care?

The term “artificial aquatic ecosystems” (along with similar terms such as “human‐made ecosystems,” see Clifford and Heffernan 2018; Koschorreck et al. 2020) is relatively new. Describing an ecosystem as “artificial” would likely sound oxymoronic to the originators of the ecosystem concept, who saw their discovery of landscape‐scale energy‐species relationships (“ecosystems”) as a call to protect specifically “natural” places unmodified by humans (Odum 1959). However, here we aim to show that the emergence of terms like AAE, that center the human role in the ecosystem, is not naive or unnecessary rebranding. It is instead a call for scientists to come to terms with the growing number of waterbodies where human activities play a defining, rather than modifying, role in their existence.

**FIGURE 1:**
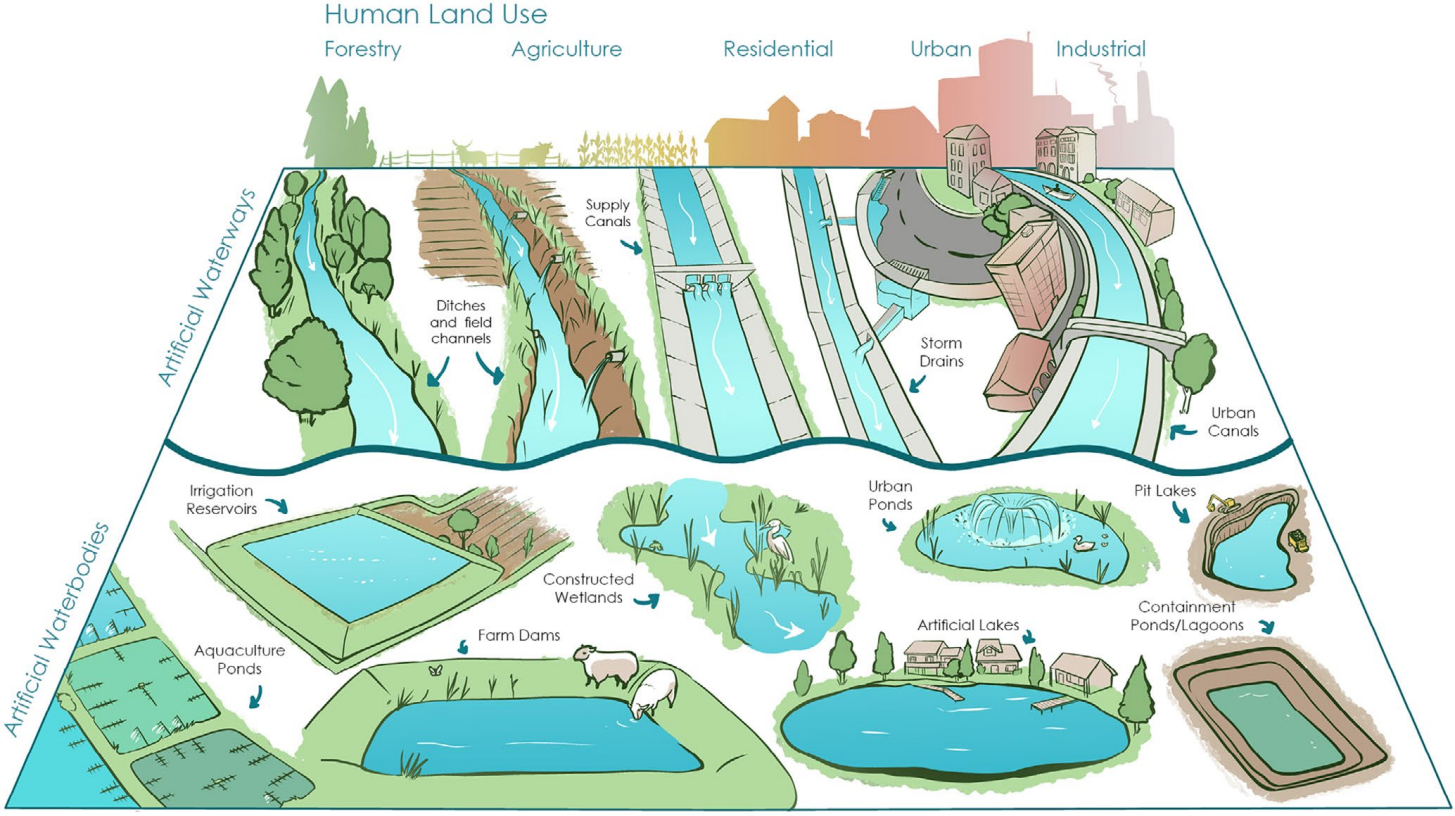
Artificial aquatic ecosystems are created to retain (“waterbodies”) or transport (“waterways”) water across the breadth of rural and urban landscapes to meet diverse human needs. Figure by Rhea Ewing.

**FIGURE 2:**
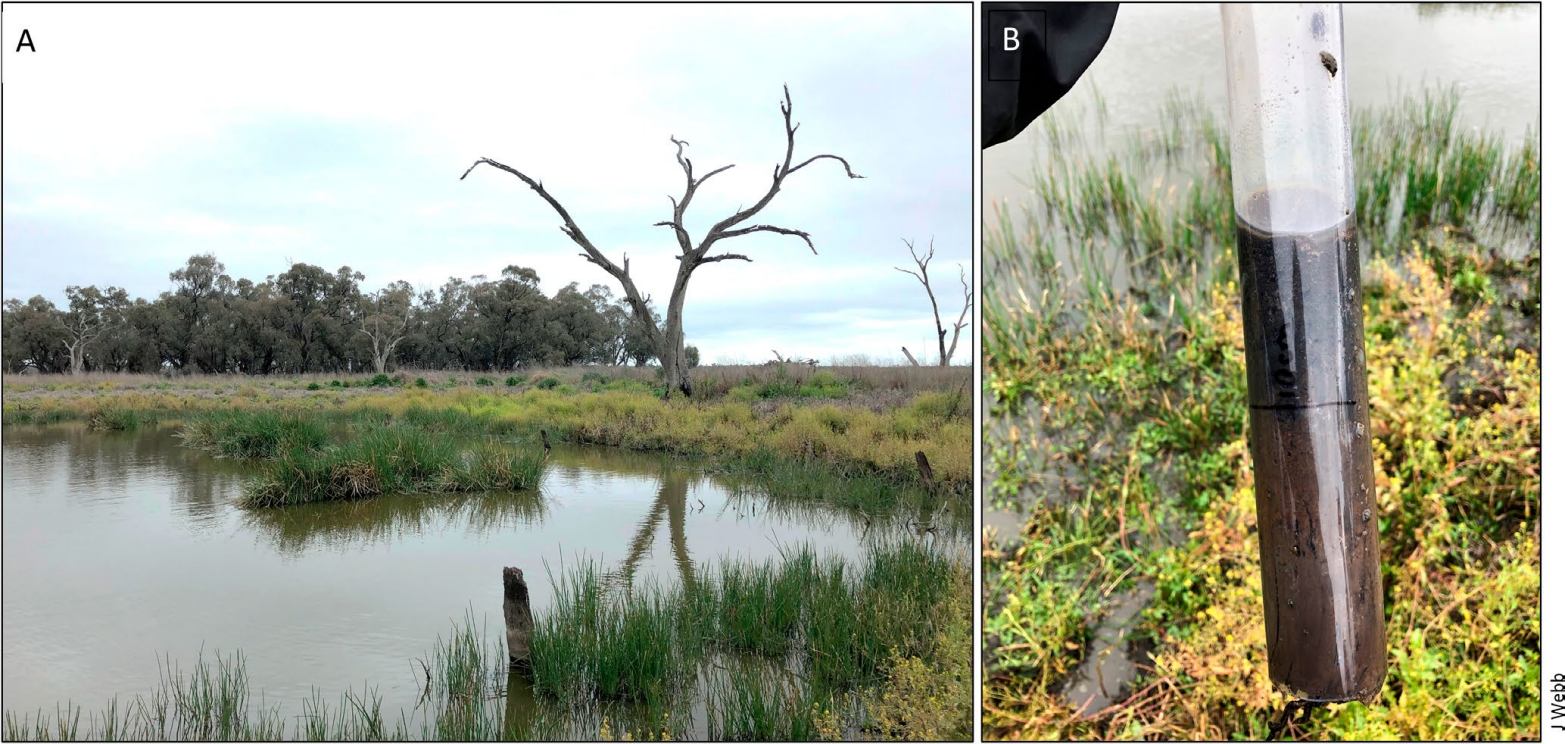
The lines between natural and artificial can be blurry for aquatic ecosystems. (A) A century‐old farm dam that was dug into a depressional wetland located on an irrigated crop farm in semi‐arid New South Wales, Australia. (B) How carbon‐rich sediments continued to accumulate in the bottom of the dam after construction, representing an important ecosystem service.

The structure and form of the world's waterbodies increasingly reflect human engineering rather than natural hydrologic or geomorphic processes (e.g., Grill et al. 2019). The growing ubiquity of dammed rivers or eutrophic lakes fits cleanly into conceptual models that include humans as a “state factor” (Amundson and Jenny 1997), a “disturbance” (Holling 1973), or an “invasive pest” (O'Neill 2001) in ecosystem development. For example, much freshwater research is done on “natural” lakes, streams, or rivers, but many of these systems are subject to a high degree of human impact—or artificiality (see Box 1). Increasingly, the waterbodies we encounter do not fit comfortably into traditional frameworks that position human impacts as one of many contributing factors to ecosystem function (Figure 1). These include a diverse range of systems from lakes dug into agricultural fields isolated flowing drains in what was once a boggy wetland, concrete channels to direct urban runoff, and strategically constructed waterbodies known as pit lakes/lagoons to manage industrial pollution. Distinguishing “artificial” from “modified” (“novel” from “hybrid,” sensu Hobbs et al. 2014) as “ecosystems without an aquatic historical precedent” will help create coherent and meaningful freshwater science and conservation strategies. Defining an “artificial” classification to distinguish waterways whose origin is fully anthropogenic is consequential because it means that AAEs have no “natural” state that can be used as a baseline for current or projected conditions. In AAEs, humans have reset, rather than merely disturbed or influenced, the trajectory of ecosystem development (Figure 2).

BOX 1 **Artificial Aquatic Ecosystems are Globally Ubiquitous—and Distinguishing Them From Other Human‐Modified Freshwaters Matters for Science and Management.**

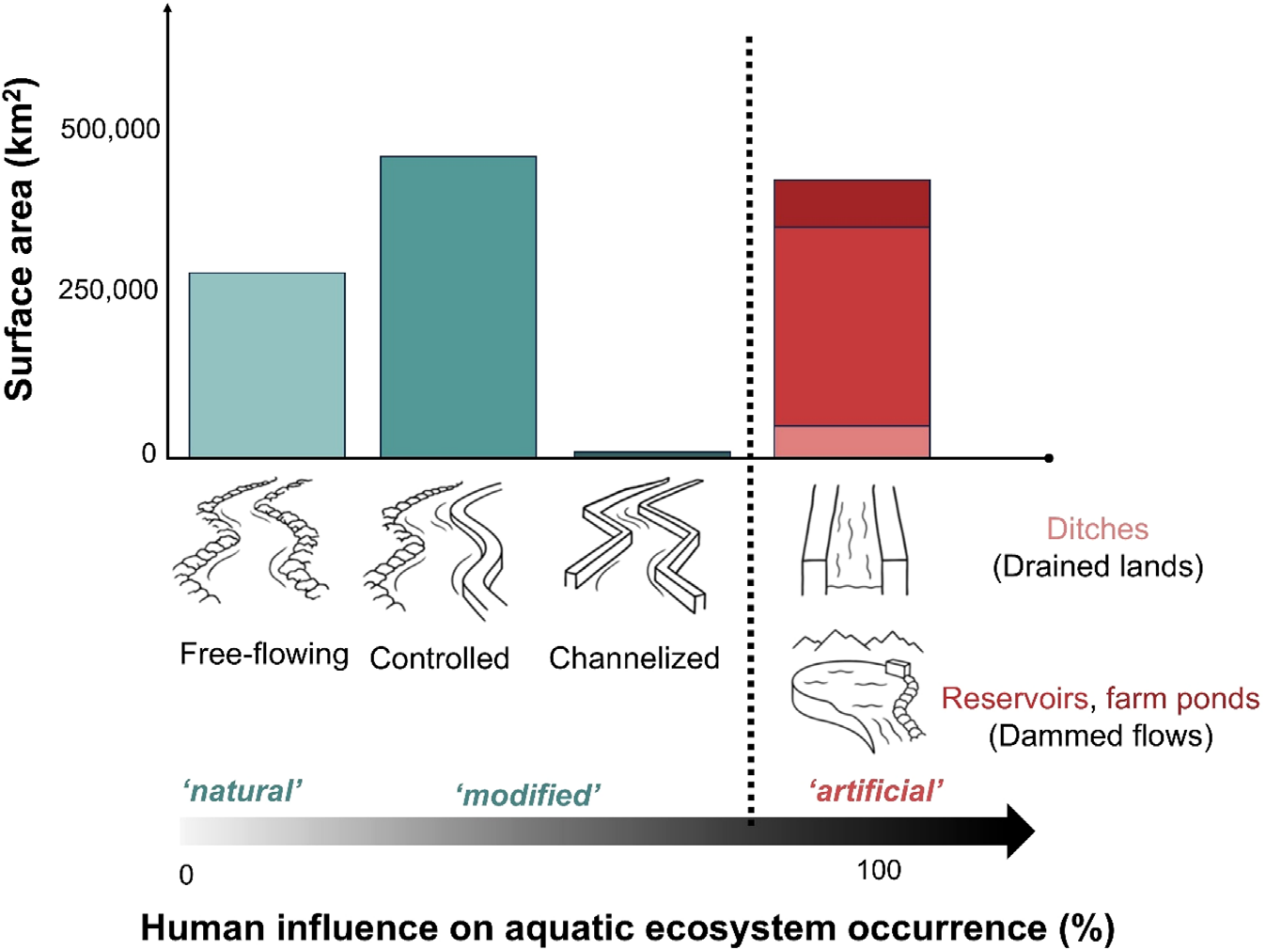

Humans influence the form and function of aquatic ecosystems: rivers are often conceived as existing on a gradient of human influence shifting them from “natural” (e.g., free‐flowing rivers) to “modified” (e.g., controlled and fully channelized rivers). We propose that “artificial aquatic ecosystems” (AAEs) are the end‐members of this spectrum, where humans are not modifiers but rather the originators of the waterway (flowing or lotic ecosystems: ditches and canals) or waterbody (standing or lentic ecosystems: farm dams and reservoirs) in the landscape. In AAEs humans either create an aquatic system where there was none previously (e.g., constructing a farm pond by collecting runoff at the base of a hillslope), or change the structure of the aquatic ecosystem so fundamentally that “before‐after” comparisons are rendered meaningless (damming a river to create a lake). The growing importance of AAEs as freshwater ecosystems is emphasized by the large proportion of aquatic surface area that they encompass. As humans continue to modify landscapes and climate change disrupts patterns of water flows and storage, AAEs will play an increasingly consequential role in sustaining society and freshwater ecosystems. The figure above illustrates this by comparing estimates of the global surface area of the remaining free‐flowing waterways (286,010 km^2^: 37% (Grill et al. 2019) of the total 773,000 km^2^ of streams and rivers (Allen and Pavelsky 2018), controlled (482,294 km^2^: 63% (Grill et al. 2019) of the 773,000 km^2^ of streams and rivers that are no longer free flowing (less those classed as channelized), channelized (4696 km^2^: assuming waterways cover the mean 0.58% of surface area (Allen and Pavelsky 2018) across the 809,664 km^2^ of urban areas (Li et al. 2020), and that these urban streams are channelised or highly modified (Walsh et al. 2005), ditches (53,530 km^2^: following estimates from Peacock et al. (2021a, 2021b), reservoirs (315,700 km^2^: satellite‐based estimates for large dams (Lehner et al. 2025), and farm ponds as small impoundments or dams (77,000 km^2^: following estimates from Saunois et al. (2025). These estimates around human modification of waterways and waterbodies are illustrative but highly uncertain—of those highlighted here only the spatial coverage of reservoirs has been validated.

## 3 Current Trends in the Literature

**FIGURE 3:**
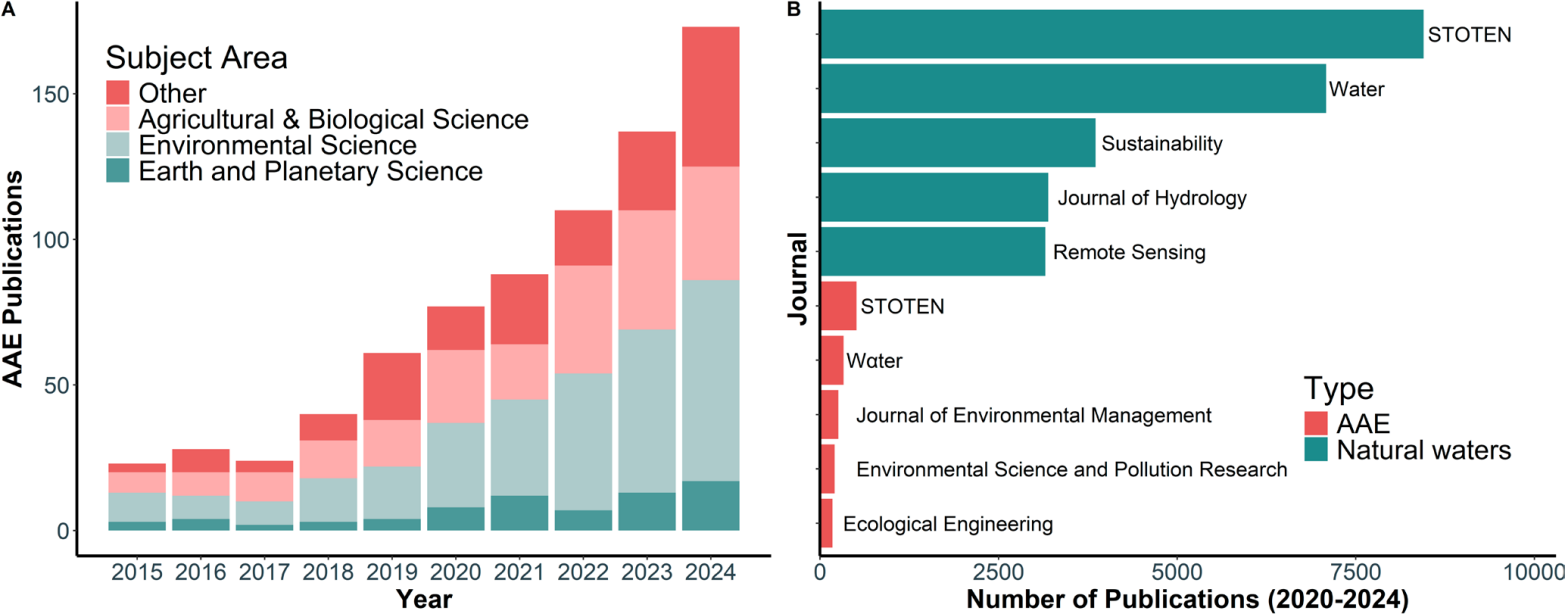
(A) The total number of publications on artificial aquatic ecosystems (AAE) in the past 10 years with bar color indicating subject area, and (B) the top five journals based on publication quantity for studies on natural aquatic ecosystems and artificial aquatic ecosystems since the seminal AAE papers by Saulnier‐Talbot and Lavoie (2018), Clifford and Heffernan (2018) and Koschorreck et al. (2020) were published. STOTEN stands for *Science of the Total Environment*.

“Hidden Treasures” (Koschorreck et al. 2020) and “Artificial Aquatic Ecosystems” (Clifford and Heffernan 2018) contextualized the otherwise fragmented studies on human‐made aquatic systems into a new area of research. Since the work of Saulnier‐Talbot and Lavoie (2018), Clifford and Heffernan (2018) and Koschorreck et al. (2020), the number of publications recognizing artificial aquatic ecosystems have doubled every three years (Figure 3A; see Appendix S1 in literature search detail). With this growth, the subject areas of AAE studies are diversifying (Figure 3B). The top cited AAE publications in the fields of Agricultural and Biological Science, Environmental Science, and Earth and Planetary Science recognize the role AAEs have in global carbon emissions, agroecosystem climate change adaptation, and freshwater biodiversity conservation (Rosentreter et al. 2021; Aguilera et al. 2020; Keller et al. 2021; van Rees et al. 2021). Research on AAEs is evolving towards a place that recognizes their importance in diverse disciplines and the environment.

However, AAEs remain underrepresented in the scientific literature relative to their natural counterparts. AAE studies represent just 5%–7% of natural aquatic ecosystem studies, as evidenced by the top five journals for each (Figure 3B). When we look at publication to global river surface area ratio based on values in Figure 3B and Box 1, a bias still exists towards natural ecosystems, with 1 publication to every 30 km^2^ of natural flowing waters vs. 1:300 for AAEs. Despite the numbers, research on AAEs is growing in the broader environmental science journals (e.g., *Science of the Total Environment* (STOTEN) and *Water* being the top two for both natural and artificial aquatic ecosystems), but not in the top “aquatic science” journals. For instance, keyword hits on the different types of AAEs in popular society journals *Limnology and Oceanography* and *Journal of Geophysical Research: Biogeosciences* between 2020 and 2024, show AAE publications only represent 1%–2% of equivalent natural aquatic ecosystem studies (Table S1).

The scientific lens struggles to focus on AAEs. Urban canals are termed “human‐impacted rivers”, ditches as “agricultural rivers” or “agricultural streams”, and completely artificial waterbodies as “urban lakes” (Tank et al. 2021). This naturalization of AAEs obscures the role of humans within freshwater ecosystems and tends to leave AAEs at the margins of freshwater research. Arguments about whether an aquatic ecosystem is “artificial” or “natural” are not just a question for theorists: legislations such as the United States Clean Water Act (2023) explicitly exclude AAEs from water quality regulation, while *only* AAEs are counted as contributing to global anthropogenic freshwater methane emissions (IPCC 2019). In the following sections, we argue that AAEs require scientific attention in order for us to continue advancing global systems knowledge and creating effective environmental policies.

## 4 Abundance and Distribution

**FIGURE 4:**
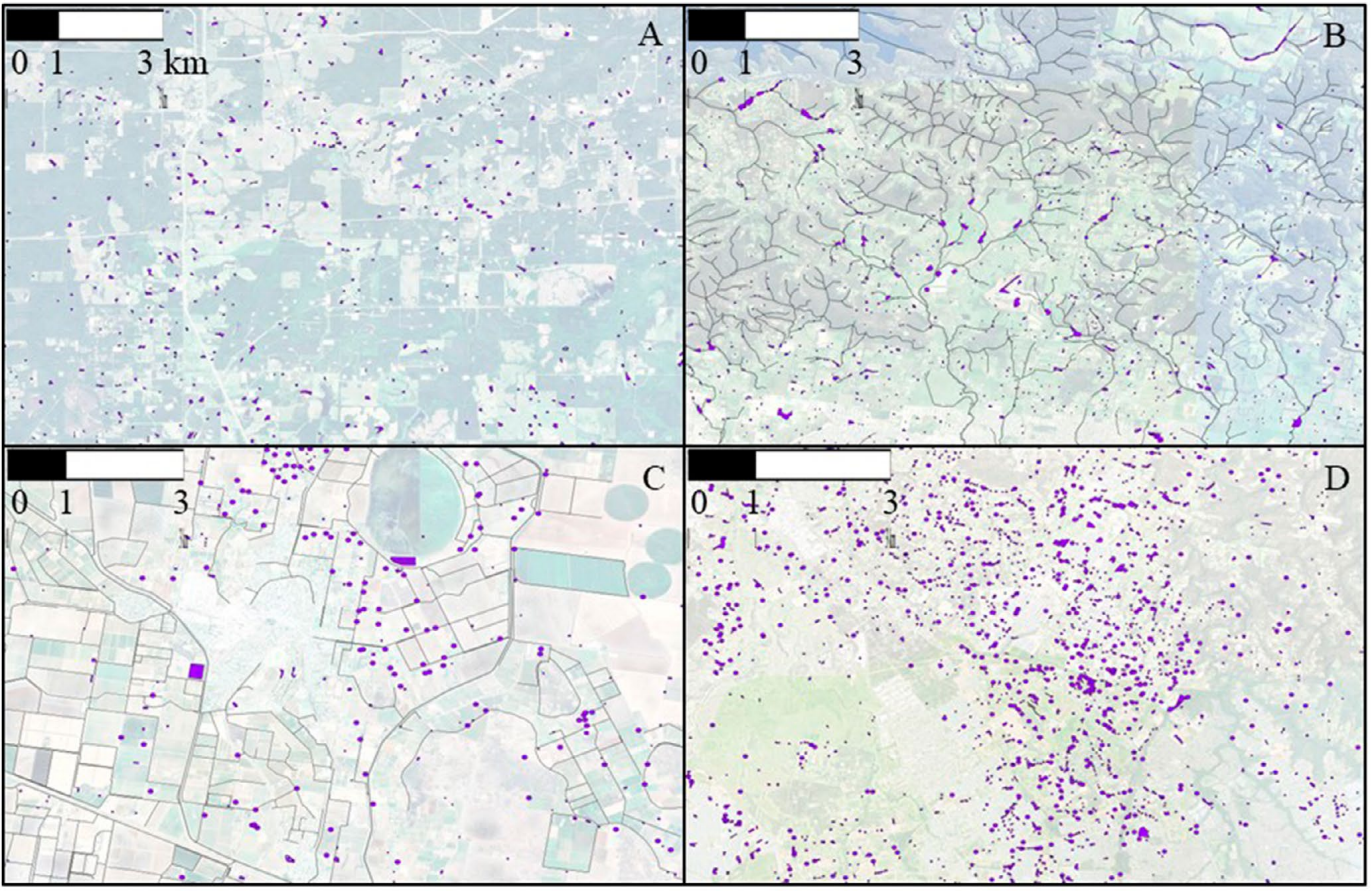
(A) The Pond Belt in the central US contains more than 2 million agricultural ponds (example near Mansfield (Louisiana, US) at 32.12134°, −93.81385°; Swartz and Miller 2021). (B) The State of Victoria (Australia) contains an estimated 450,000 agricultural ponds (blue polygons; Malerba et al. 2021) and over 7000 km of irrigation canals (black lines; Crossman and Li 2015). Example from the Yarra Valley (38.05977°, 145.48965°). (C) The Murrumbidgee Irrigation Area (New South Wales, Australia) contains 3500 km of irrigation channels (brown lines) which feed water into on‐farm dams (purple areas) known locally as storages (Kirby 2011). This estimate only includes permanent supply and drainage distribution canals and not on‐farm small channels. Example from near Narrandera (−34.60419°, 146.41602°). (D) The distribution of urban waterbodies near Beaumont in Sydney, Australia (−33.70925°, 150.93621°).

Accurately quantifying the total area and distribution of AAEs is challenging due to their high densities and relatively small sizes (figure 4; Malerba et al. 2021). The satellite images in Figure 4 identify some of these small water features within grazed, irrigation, and urban landscapes. Note that waterbodies (polygons) tend to appear more easily on maps than watercourses (lines). Some canals are substantial in width (10–50 m) due to the transport of large volumes of water for farming industries and regional cities, while drainage ditches are small but will appear much bigger on vector lines if identified as a mapping feature. Additionally, many farm ponds and ditches dry out periodically (Vaughan et al. 2008; Malerba, Wright, and Macreadie 2022), making spatial analysis of their inland surface water area inherently difficult without temporally resolved satellite image mapping techniques. Using infrastructure records or other publicly available regulatory documents can provide an alternative approach to identify and map the number, location, and magnitude of AAEs (e.g., Sinclair et al. 2020; Rains et al. 2023; Rains et al. 2025).

Initial attempts to estimate the global presence of lentic waterbodies used size‐area relationships, which extrapolate global values based on smaller‐scale patterns between surface areas and abundance (Downing et al. 2006). For both natural and artificial water bodies, the most common approach to model the size distribution at large scales is by assuming a Pareto distribution (McDonald et al. 2012). This approach is justified under a model in which small waterbodies merge into larger ones with greater inundation extent, revealing a predictable pattern (Stachelek 2023). Size‐abundance relationships suggested that larger water impoundments cover more than their smaller counterparts (Downing et al. 2006; McDonald et al. 2012; Smith et al. 2002). For instance, large dams and reservoirs span ~260,000 km^2^ globally, while farm ponds cover ~77,000 km^2^ (Downing et al. 2006). Yet, recent studies contest this “one‐size‐fits‐all” approach because of unaccounted variation and bias in size‐shape relationships of artificial systems among landscapes (Seekell and Pace 2011).

Beyond size‐abundance relationships, the era of high‐resolution satellite imagery has improved area estimates of freshwater systems. Thousands of dams can now be mapped with unprecedented detail (Mulligan et al. 2020; Verpoorter et al. 2014). However, the picture gets fuzzier when mapping small AAEs—such as ponds, channels, and ditches. Mapping the number and distribution of these AAEs, which can vary between years, remains challenging (Figure 4C) but promising methods now exist (Lidberg et al. 2023). While no global dataset exists for these smaller features, regional and continental efforts in places like the United States (Sinclair et al. 2020) and Australia (Malerba et al. 2021) provide valuable insights. For example, satellite imagery has helped to understand the growth rate of farm dams in Australia, which shows a varied growth rate from 0.8% to 3.2% per annum (Malerba et al. 2021). These techniques can even map water fluctuations within farm dams, providing insights into the reliability of these systems to store water under climate change (Malerba, Wright, and Macreadie 2022).

## 5 Contribution to Freshwater Research

The following subsections consider some of the common AAEs presented in Figure 1 and how they are considered in research and policy. Our goal here is not to provide an exhaustive description of all AAE types, but rather, draw on case studies to demonstrate the advantages and opportunities of improving our scientific understanding of these systems. We include a brief synopsis of historical research angles, ecosystem services and disservices, scientific understanding, and policy for various lotic and lentic AAEs. These include artificial channels, which covers entirely human‐made channels that did not naturally exist as rivers (e.g., canals and ditches) and small artificial waterbodies (typically < 0.01 km^2^) that represent lentic systems that have formed from the impoundment of small creeks or ephemeral waterways (farm dams, constructed wetlands), as well as the creation of new waterbodies for water storage (e.g., irrigation dams, dugouts, urban ponds).

### 5.1 Urban Channels

**FIGURE 5:**
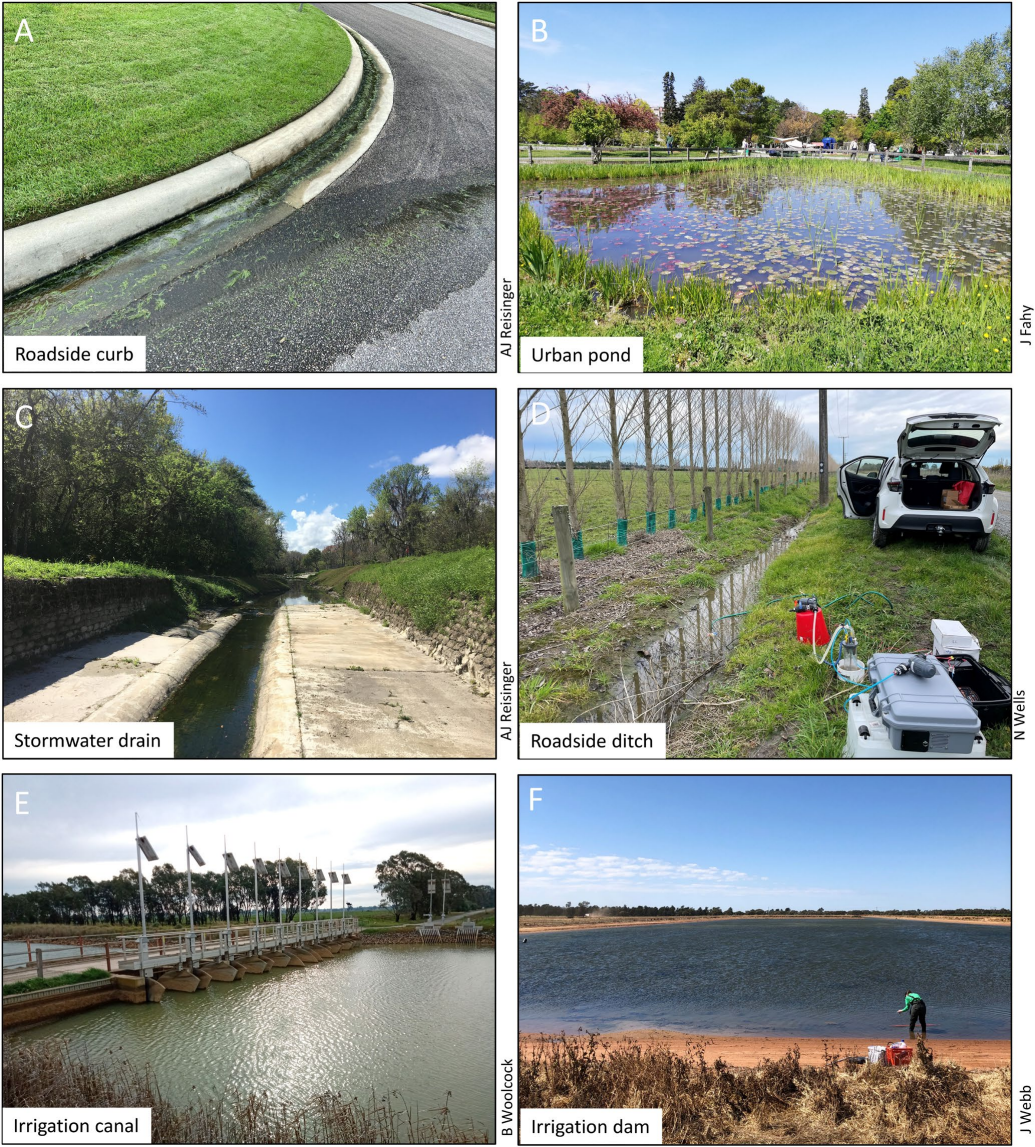
The varying scales of artificial aquatic ecosystems. Some are so ubiquitous that they exist right outside the home (roadside curbs; A), in local parks (urban ponds, B), and along road lines (stormwater drains, C; and roadside ditches, D). While others can be in isolated locations but as large as rivers and lakes, such as 100–1000s km long water supply canals (E) and farm storage dams (14 ha irrigation dam for horticultural production, F).

The hydrological, chemical, and biological conditions of urban streams have been studied for decades. Our understanding of the impacts of urbanization on streams led to the conceptualization of the urban stream syndrome (Walsh et al. 2005). Although originally formulated to describe how urbanization affects natural streams, the role of artificial urban channels and their response to urbanization has been long identified as a research priority (Wenger et al. 2009). Artificial urban channels include highly artificial channels such as gutters on houses (Fork et al. 2018) and roadside curbs (Bratt et al. 2017), more “naturalized” artificial channels such as roadside ditches (Tatariw et al. 2021) and canals (Dehini and Gomes 2022), or natural streams that have been modified via channelization, altered geomorphology, or various ecological restoration techniques (Figure 5A,C,D).

Although there is a relatively long history of urban stream ecology and a broad focus on various ecological concepts in urban streams, there has been limited focus on artificial urban channels. Research on artificial urban channels has focused more on hydrological (e.g., Anim et al. 2019) and chemical (e.g., Gallo et al. 2012) dynamics rather than the interrelated ecological processes. For example, their original function in urban areas is to increase stormwater runoff so as to mitigate possible flooding, but in doing so many concrete urban channels become prime conduits for contaminants (Taylor and Owens 2009). This ecosystem disservice of stormwater pollution has been widely recogniz ed. In water sensitive water design legislation since the 1990 s (US EPA 1996; Whelans et al. 1994). Similar to other areas of green infrastructure, there is growing evidence that constructing or restoring artificial channels positively impacts biota (Reid and Church 2015). Despite this increasing recognition of their potential ecological importance, there remains a lack of research focused on ecological dynamics of urban artificial channels beyond design considerations. These artificial channels could serve as valuable opportunities to answer fundamental questions related to ecological dynamics of lotic ecosystems while also providing important information to managers hoping to enhance ecosystem services provided by urban channels.

### 5.2 Irrigation Channels

Irrigation channels or canals transport water throughout arid regions for agricultural needs. These waterways are characterized by long straights with sharp turns, with some systems stretching hundreds of kilometers in length (Figure 4C). In many irrigation channels (e.g., in southeastern Australia, and in irrigation ditches in agricultural European peatlands) high flows occur in summer and low flows occur in winter, while a natural river in the same region would experience the opposite flow regime. However, as many rivers are now also heavily regulated, their flows may resemble those of irrigation waterways (figure 5E; McMahon and Finlayson 2003, Peacock et al. 2017).

The potential ecosystem services of irrigation channels have been seldom investigated, with most attention devoted to water efficiency or entrainment of organisms in these waterways (King and O'Connor 2007; Ooi and Weyer 2008). Irrigation channels are becoming important refuge habitats in regions that are experiencing an increase in drought frequency and a reduction in natural wetlands, given they provide a regular source of water compared with natural waterways (Chester and Robson 2013). With human intervention, these AAEs could become sources of organisms to boost natural populations. For example, the Victorian Fisheries Authority (VFA 2021) salvaged close to 1700 native fish from irrigation channels in northern Victoria, Australia.

Management decisions of irrigation channels rarely consider the ecological functions of these human‐made systems (Pereira et al. 2002). The growing impact of water competition has led to innovative solutions being explored to improve the sustainability of irrigation infrastructure. One example is a project trialing solar panels over canals to reduce water losses and weed growth, which is currently being tested in California (Bales 2022). Yet due to their primary function being water delivery and efficiency, the environmental impacts on the freshwater ecology of the canals are rarely part of the discussion. Nevertheless, there are cases in Australia where management of irrigation channels is temporarily shifted to benefit natural freshwater systems, including the delivery of oxygenated water to natural waterways during hypoxic blackwater events (Watts et al. 2018).

### 5.3 Urban Ponds

Urban ponds represent another type of AAE designed for a variety of purposes, from retaining stormwater to improving esthetics (Figure 5B). They deliver a wide array of ecosystem services, including water storage and purification, societal health benefits (i.e., urban “blue spaces”), as well as biodiversity provisioning. However, the effectiveness of these various societal contributions is rarely assessed, and the desired outcomes may not be achieved. For example, nutrient concentrations in effluents from water treatment ponds often exceed regulations (Manzo et al. 2020), potentially due to internal biogeochemical cycles within the ponds (Goeckner et al. 2024), which impacts the credibility of these ponds for controlling water quality.

Research appears split between engineering journals, with a focus on stormwater ponds and nutrient processes (Troitsky et al. 2019), and traditional freshwater journals, with a focus on their role for biodiversity and human well‐being (Oertli and Parris 2019). For example, much of the ecological research about urban ponds revolves around their biological richness relative to more natural ponds (e.g., Hill et al. 2017; Perron and Pick 2020). Urban ponds are sometimes viewed as poorer in terms of biodiversity (Noble and Hassall 2015), yet some stormwater ponds host similar richness to natural ponds (Hassall and Anderson 2015). Many urban ponds are characterized by high nutrient inputs due to their role of storing stormwater runoff, and are often inhabited by exotic invasive species valued for their aestheticism (Sinclair et al. 2020). These effects on biodiversity reflect the wide differences in environmental conditions between urban ponds and natural waterbodies in terms of their design, water chemistry, connectivity and biological communities.

An integrative view of urban pond research would help guide their effective design and management. Urban ponds often lack multifunctionality despite their potential (Oertli et al. 2023), and more research is needed to ensure that multiple co‐benefits of a pond will translate into practice. Existing success stories include, for instance, a demonstration project in Lystrup, Denmark, where the creation of a pond in a public park led to effective flood risk reduction alongside biodiversity objectives and community involvement (Biggs et al. 2024). In Geneva, Switzerland, a network of urban ponds with different purposes has been shown to collectively deliver positive public perception and to host relatively diverse pond communities (Oertli et al. 2023). Others have found similar social expectations for urban stormwater ponds in Florida, which are appreciated for the flood control they provide, but are also valued for ecosystem services related to esthetics, property values, and biodiversity provided by ponds (Monaghan et al. 2016).

### 5.4 Farm Ponds and Reservoirs

Farm ponds are constructed by impounding small streams and/or excavating depressions to support crop and livestock watering, fish and game production, and recreation (McMurry 2020; Webb et al. 2019). Globally, farm ponds represent the novel addition, expansion, or replacement of lentic waters lost due to land use conversion and wildlife management (Berg et al. 2016). Depending on the origin of their construction (draining of a wetland vs. creating a new waterhole on barren land), some farm ponds provide high conservation value as drought refuge for aquatic fauna, boosting regional biodiversity (Usio et al. 2013). Determining whether these artificial aquatic ecosystems function differently from natural ponds is complicated by their poor representation in the scientific literature relative to larger waterbodies and the lack of distinction among pond origins in published works (Downing 2010; Richardson et al. 2022).

Understanding the cumulative impacts of farm ponds requires an understanding of their natural analogues. Farm ponds have been explicitly identified as hotspots of GHG emissions (Grinham et al. 2018; Ollivier et al. 2019) and generally have higher emissions than their natural counterparts (Peacock et al. 2021a, 2021b). Both natural and constructed ponds can receive high terrestrial organic matter loads and have high sediment area to water volume ratios but, because of intensive surrounding land use, inputs of nitrogen and phosphorus will generally be higher in constructed ponds. Additionally, farm ponds are often younger than natural ponds, which may mean that more labile organic matter remains for microbial degradation, resulting in GHG production (Jensen et al. 2023). Because farm ponds are constructed, their bathymetry frequently differs from that of natural ponds. Depth is a key determinant of pond mixing regimes, and because mixing can impact GHG production and emission (Holgerson et al. 2022), we may expect differences in the magnitude and controls of emissions between system types. Supporting this concept, studies of natural and constructed lentic waterbodies, including farm ponds, have found different environmental drivers of emissions (Deemer and Holgerson 2021; Jensen et al. 2023).

The artificial characteristics of farm ponds relating to differences in their management, land use, and age can create opportunistic experiments to help advance the science of freshwater lentic systems. For example, constructed ponds have been used as model ecosystems to understand the role of small lentic freshwaters in the global carbon budget and how management actions influence burial rates (Holgerson et al. 2024; Ljung and Lin 2023). Their heightened ability to trap sediments, accumulate organic matter, and generate GHGs has prompted interest in the role of artificial ponds in climate mitigation strategies (figure 5B; Cuenca‐Cambronero et al. 2023). Ultimately, these lines of inquiry could enhance our understanding of ecosystem states and processes in both natural and artificial aquatic ecosystems.

### 5.5 Constructed Wetlands

Constructed wetlands (CWs) are engineered waterbodies designed to treat and retain runoff from a variety of settings: urban stormwater runoff, industrial discharges, agricultural runoff, and mining discharge (Malaviya and Singh 2012). They are designed to mimic the functionality of natural wetlands to treat contaminants accumulated from catchment areas through biological, chemical, and physical processes (Usharani and Vasudevan 2016), though with a more systematic and predictable outcome in comparison to natural wetlands (Malaviya and Singh 2012).

Since the earliest implementation of constructed wetlands, few have been studied from an ecological perspective (e.g., Lacki et al. 1991). There has been increasing research interest in the functionality, and potential multifunctionality, of constructed wetlands from the freshwater sciences community (e.g., Hambäck et al. 2023). Whereas much of the early literature was focussed on design and basic treatment performance aspects, with an engineering leaning (e.g., Gersberg et al. 1986; Reuter et al. 1992), much of the current research takes a mechanistic approach to nutrient and contaminant cycling within constructed wetlands, and there is much work around unpacking the interactions between the microbiological, macrophytic and geochemical processes at play (e.g., Zhao et al. 2024).

This interest from a broad range of fields is unsurprising: Because of their design and functionality, CWs represent both the “contaminated” scenario (at their inlet) and the “remediated,” if not pristine, scenario at their outlet, where they discharge water to the natural environment downstream. As such, a single wetland can be considered as both an example of an extreme, but also a “conventional” freshwater system. Although a unique opportunity from a research perspective, this is a limitation from a management and policy perspective. For example, in Australia, there are performance guidelines for CWs with respect to suspended solids, total nitrogen, total phosphorus, and litter (Victorian Stormwater Committee 1999). To evaluate metal contaminants, it is necessary to defer to the Australian and New Zealand Guidelines for Fresh and Marine Water Quality (ANZECC and ARMCANZ 2018) and the National Environment Protection Measure (NEPM 1999). However, both of these guidelines require an understanding of the acceptable level of ecological impact before threshold concentrations can be established. There is a clear opportunity to establish sediment and water quality guidelines specific to CWs as they represent the “last line of defense” between stormwater and natural water bodies.

## 6 A Blind Spot in Environmental Policy

Artificial aquatic ecosystems, and particularly small waterbodies, have largely been excluded from environmental policy (Clifford and Heffernan 2018; Koschorreck et al. 2020). For example, the European Union's Water Framework Directive, which stipulates that waterbodies must be “healthy” from a physical, ecological and chemical perspective, includes lakes, rivers, estuaries and coastal waters, but excludes ponds and ditches (Williams et al. 2004; Hill et al. 2017). In the US, recent revisions to the definitions of “waters of the United States” (i.e., those waterbodies that fall under the remit of the Clean Water Act) still omit the majority of small AAEs (EPA 2023). Exceptions do exist, such as the Australian and New Zealand Environment Conservation Council (ANZECC) for irrigation channel water quality. However, these guidelines were developed for crop performance (i.e., industry guidelines) and not ecosystem health, in that thresholds for nitrogen (N) and phosphorus (P) are high; 25–125 mg L^−1^ and 0.8–12 mg L^−1^ for N and P, respectively (ANZECC and ARMCANZ 2018), which are effectively hypereutrophic (IPCC 2019). The omission from water quality policy results in a lack of monitoring, and therefore understanding, of the state of artificial aquatic ecosystems. Even countries with well‐funded, long‐running monitoring programs of surface waters neglect their artificial aquatic ecosystems. For example, the Swedish National Monitoring routinely measures ~5000 lakes and ~100 watercourses, but this includes only a handful of forest or agricultural ditches (Fölster et al. 2014; Kyllmar et al. 2014), and constructed ponds are absent altogether. This is despite Sweden having developed regional targets for increasing the number of CWs to reduce eutrophication and enhance biodiversity (Geranmayeh et al. 2023).

Greenhouse gas emissions from AAEs are partially accounted for in climate policy. The IPCC now has guidelines for methane emissions from ditches/canals and constructed ponds (including for wastewater), but emission factors are a global “one‐size‐fits‐all”. Carbon dioxide emissions from small artificial waterbodies remain unaddressed. Emissions of the extremely potent GHG nitrous oxide are partially considered, but IPCC emission factors do not distinguish between artificial and natural aquatic ecosystems (Webb et al. 2021). More field measurements are needed in order to increase the evidence base and refine emission factors.

Future management of artificial aquatic ecosystems should consider the multiple and diverse ecosystem services that artificial aquatic ecosystems can provide, rather than focusing myopically on the purpose they were designed for. For instance, irrigation channels and drainage ditches are generally viewed only through a hydrological lens, and thus the flora and fauna that inhabit these waterways are not considered in management plans or guidelines, despite them being potentially valuable habitats (Carlson et al. 2019; Clifford and Heffernan 2018). Similarly, farm ponds and agricultural wetlands can provide a myriad of other ecosystem services in addition to water storage, including biodiversity provision, recreation (bird‐watching, hunting, ice‐skating), and esthetics (Geranmayeh et al. 2025). Provided that the originally intended functions of these waterbodies are maintained, it may be feasible to adjust management and policy to provide ecosystem service co‐benefits.

## 7 Advancing Research in Freshwater Science and Policy

Artificial aquatic ecosystems have perhaps been overlooked by freshwater scientists and policy makers because they fall somewhere between infrastructure and a natural resource. Therefore, the scientific study and regulatory frameworks of these two “categories” operate in isolation. This is complicated by the fact that they involve a mix of uses and stakeholders, from municipalities to private owners, with no common framework. Although designed for human service, many AAEs actually support multiple ecosystem services, representing an exciting opportunity in freshwater science research and policy. To achieve these goals, we conclude by highlighting some important gaps to be addressed to refocus our view of AAEs.

### 7.1 Reframe

Reframing AAEs as systems that can support important ecosystem values rather than utilitarian bodies of water may encourage greater buy‐in from policy makers, funders, researchers, and landholders in the management and understanding of these systems. Many AAEs currently represent the final line of defense between environmental stressors such as water use by primary industry, eutrophication, and pollution, and natural freshwater bodies like lakes, streams, and rivers. When functioning as designed, they buffer, reduce, and, in some cases, completely mitigate the impacts of these stressors. But they can only play this role if they are properly maintained, and these roles can only be optimized with ongoing research.

### 7.2 Incentivize

Investment should come from governments, either through policy or incentives to encourage landowners and councils to manage AAEs not just for their primary service but for the multiple services they offer. For example, small AAEs like ditches and ponds contribute to a country's anthropogenic emissions and some groups are advocating for carbon credit payments to farmers for adopting methane mitigation practices in their farm dams (Malerba, Lindenmayer, et al. 2022). With the current lack of regulation to incentivize such changes, we need to bring together experts in the water sector to set out a list of priorities for the water quality in these systems. Initial investment will likely originate from localized projects with community groups, interested landowners, and innovative organizations in the water sector, as we have seen with a stakeholder‐driven interdisciplinary agricultural ditch restoration project, Canterbury Waterway Rehabilitation Experiment (CAREX), in New Zealand (Collins et al. 2020). Another example is that of Aarhus Municipality, in Denmark, which implemented rainwater retention basins, dykes and swales as Nature‐based Solutions to combine climate adaptation, recreational use, stakeholder involvement, and biodiversity objectives (Biggs et al. 2024).

### 7.3 Monitor

Artificial aquatic ecosystems are often easily accessible, and in locations where power supplies and internet can be readily sourced. This makes them ideal for low‐cost, long‐term monitoring studies as well as short‐term investigations. The urban setting of some AAEs can also make them prime subjects of citizen science projects. For example, volunteers helped map urban ponds and collect habitat data in England through the “Urban Pond Count” project (Freshwater Habitats Trust, n.d.). While natural lakes offer insights into past conditions and changes in the landscape and climate (Adrian et al. 2009), AAEs represent a window into future conditions that may be extreme or uncommon now. Investment into harmonized long‐term research of AAEs, similar to the NSF Long Term Ecological Research and NO‐L2 schemes in the USA and Norway, is an aspirational goal. Until then, prioritizing systems that are truly on the “front line” of freshwater pressures, like downstream pollution, eutrophication, water availability for primary industries, and threatened species, will have the greatest return on investment.

### 7.4 Integrate With the Natural Sciences

Ultimately, we call for improved recognition of AAEs as fundamental components of the freshwater sciences. As a first step, we need to recognize AAEs as functioning units (pools and fluxes) in the water cycle, as they are not included in traditional or modern‐day diagrammatic examples, despite human freshwater appropriation now equaling half of the global river discharge (Abbott et al. 2019). The widespread presence of AAEs in the modern hydroscape (e.g., Rains et al. 2023) places artificial and natural waters as interconnected systems across spatial scales, meaning that AAE research efforts are a natural extension to freshwater research. Secondly, we stress the need to explicitly include AAEs into inland water biogeochemical cycles where planetary boundaries have been breached, including carbon and nitrogen, where AAEs have a role to play in either exacerbation or mitigation of these elements. Put simply, better management of AAEs to mitigate downstream pollution will lead to better outcomes for natural aquatic ecosystem health. Progress in this area includes AAEs recently being included in a revised framework for quantifying landscape carbon budgets (Casas‐Ruiz et al. 2023), in IPCC guidelines (IPCC 2019), and in some country‐wide carbon budget estimates as anthropogenic aquatic emissions (van Baren et al. 2024).

Finally, freshwater scientists have a role to play by acknowledging the importance of these systems and investing in interdisciplinary collaboration. Change starts at education, so we encourage those that teach any aspect of freshwater science to update materials and field studies to include AAEs. We challenge all science societies that focus on inland waters to better facilitate inclusion of unnatural freshwaters into their community. This could be by recognizing excellence in this area of research through awards, broadening journal aims, providing seed funding for AAE research, or establishing an AAEs working group to create new initiatives and revise society strategic plans.

## Author Contributions

**Jackie Webb:** conceptualization (lead), data curation (equal), funding acquisition (lead), project administration (lead), visualization (equal), writing – original draft (equal), writing – review and editing (equal). **Ellen Moon:** conceptualization (supporting), data curation (equal), writing – original draft (equal), writing – review and editing (equal). **Julie C. Fahy:** data curation (equal), writing – original draft (equal), writing – review and editing (equal). **Shashini Fernando:** data curation (equal), writing – original draft (equal), writing – review and editing (equal). **Martino E. Malerba:** conceptualization (supporting), data curation (equal), visualization (equal), writing – original draft (equal), writing – review and editing (equal). **Laura Naslund:** data curation (equal), writing – original draft (equal), writing – review and editing (equal). **Mike Peacock:** data curation (equal), writing – original draft (equal), writing – review and editing (equal). **Naomi S. Wells:** conceptualization (supporting), data curation (equal), visualization (equal), writing – original draft (equal), writing – review and editing (equal). **Benjamin J. Woolcock:** data curation (equal), writing – original draft (equal), writing – review and editing (equal). **Alexander J. Reisinger:** conceptualization (supporting), data curation (equal), visualization (equal), writing – original draft (equal), writing – review and editing (equal).

## Funding

This work was supported by Future Earth Australia and Granders Trust, Seizing Opportunities Grant.

## Conflicts of Interest

The authors declare no conflicts of interest.

## Supporting information


**Figure S1:** Summary of Scopus literature search results for subject area that artificial aquatic ecosystem studies are published in between 2015 and 2024.
**Table S1:** Literature survey of natural and artificial aquatic ecosystem studies in the top 10% society journals that focus on freshwater ecosystems in the subject area of Aquatic Sciences. Publications between 2020 and 2024.
**Appendix S1:** Literature search.

## Data Availability

The authors have nothing to report.
