# Supplementary material for: Exploring Uncharted Waters: The Role of Artificial Aquatic Ecosystems in Freshwater Science
Source: Ecol Evol. 2026 Jun 3;16(6):e73777. doi: 10.1002/ece3.73777 (PMC13239765; doi:10.1002/ece3.73777)
Supplement: Supplementary file 1 — Figure S1: Summary of Scopus literature search results for subject area that artificial aquatic ecosystem studies are published in between 2015 and 2024. Table S1: Literature survey of natural and artificial aquatic ecosystem studies in the top 10% society journals that focus on freshwater ecosystems in the subject area of Aquatic Sciences. Publications between 2020 and 2024. Appendix S1: Literature search. [file ECE3-16-e73777-s001.docx]

***Supplemental Information***

**Exploring uncharted waters: The role of artificial aquatic ecosystems in freshwater science**

***Appendix A. Literature search***

Publication trends presented in Figure 3 of the main text were completed on 19^th^ Dec 2025 using an advanced literature search in Scopus. Search terms used for Figure 3A included "artificial aquatic ecosystems" OR "artificial aquatic ecosystem" OR "artificial waterbodies" OR "artificial waterbody" OR "artificial waterway" OR "human-made aquatic ecosystems” and were filtered by publication year range of 2015-2024. This returned a total of 761 publications that had mentioned at least one of these terms in all fields. From this search we chose to plot the top three subject areas (Environmental Science, Agricultural and Biological Science, Earth and Planetary Sciences), with the remaining subject areas grouped into ‘Other’. For the high-level scope of this paper, we did not go into any further refinement of our inclusion criteria or check the accuracy of the AAE definition in each study. A summary of the Scopus search results presented in Figure 3A are provided in Figure S1.


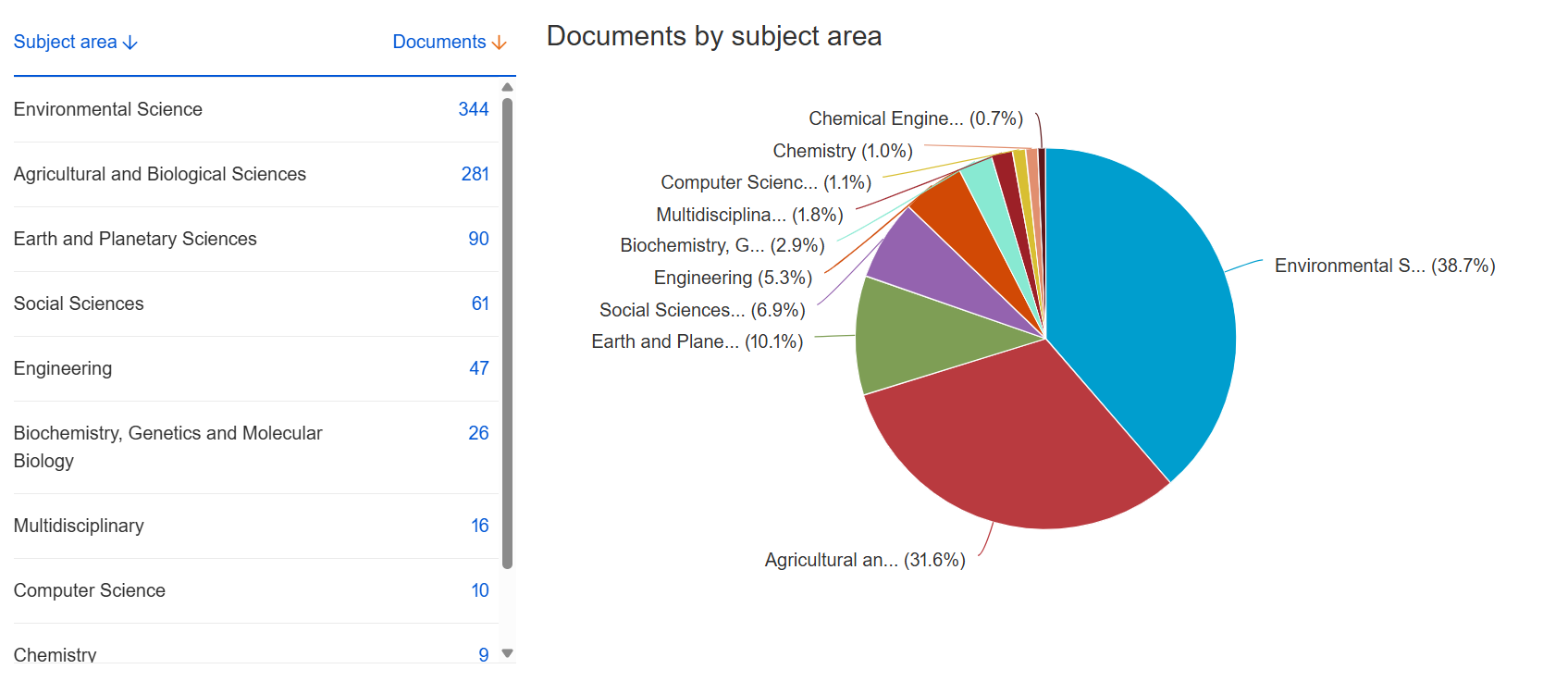


**Figure S1: Summary of Scopus literature search results for subject area that artificial aquatic ecosystem studies are published in between 2015-2024.**

To determine the most popular scientific journals that natural and artificial aquatic ecosystems published in, we chose to focus on specific types of lotic and lentic systems between the 2020-2024 period. The search terms used to compare natural and artificial aquatic ecosystem publication and journal type in Figure 3B are defined in Table S1. The search returned a total of 1,481 studies for AAEs and >25,000 for natural aquatic systems. We then completed a search focused on two of the most respected society journals for aquatic sciences during the same period using the same search terms. Results are presented in Table S1.

**Table S1: Literature survey of natural and artificial aquatic ecosystem studies in the top 10% society journals that focus on freshwater ecosystems in the subject area of Aquatic Sciences. Publications between 2020-2024.**

|  | Key words | Limnology and Oceanography | JGR: Biogeosciences |
| --- | --- | --- | --- |
| Natural aquatic ecosystems | "lake" OR "river" OR "wetland" OR "pond" OR "stream" OR "creek" | 1,326 | 915 |
| Artificial aquatic ecosystems | “farm dam” OR "agricultural reservoir" OR "dugout" OR "farm pond" OR "ditch" OR "irrigation canal" OR "stormwater pond" OR "urban wetland" OR "retention basin" OR "detention basin" OR "drainage channel" OR "supply canal" OR "main canal" OR "constructed wetland" | 13 | 20 |
